# Preoperative Zinc Deficiency as a Novel Prognostic Predictor for Postoperative Recurrence in Hepatocellular Carcinoma

**DOI:** 10.3390/nu18193147

**Published:** 2026-09-24

**Authors:** Yuki Adachi, Shingo Shimada, Shoichiro Mizukami, Tomoki Takizawa, Katsuro Enomoto, Kai Makino, Hiroki Takahata, Hiroyuki Takahashi, Koji Imai, Hideki Yokoo

**Affiliations:** Division of Hepato-Biliary-Pancreatic Surgery and Transplant Surgery, Department of Surgery, Asahikawa Medical University, 2-1-1 Midorigaoka Higashi, Asahikawa 078-8802, Hokkaido, Japan

**Keywords:** hepatocellular carcinoma, zinc deficiency, recurrence, prognosis, hepatectomy

## Abstract

**Background/Objectives**: The impact of preoperative zinc (Zn) status on the survival and recurrence of patients with hepatocellular carcinoma (HCC) after hepatectomy remains unclear. This study aimed to clarify whether preoperative Zn deficiency affects the prognosis of patients with HCC undergoing initial curative hepatectomy. **Methods**: We retrospectively reviewed 105 patients who underwent initial curative hepatectomy for HCC at our institution (2011–2018). Preoperative Zn deficiency was defined as serum Zn < 70 µg/dL. Clinical and tumor parameters were compared between patients with and without Zn deficiency. Disease-free and overall survival were evaluated using the Kaplan–Meier method, and prognostic factors were identified via univariate and multivariate Cox proportional hazards analyses. Subgroup analyses were conducted based on age, gender, and etiology. **Results**: Among 105 patients, 46 (43.8%) showed Zn deficiency (Zn deficiency group), whereas 59 (56.2%) showed normal Zn (normal Zn group). Compared with the normal-Zn group, the Zn deficiency group showed significantly lower hemoglobin, prothrombin activity, serum albumin, and smaller tumor size (all *p* < 0.05). The Zn deficiency group experienced significantly worse disease-free survival (*p* = 0.015) but no difference in overall survival (*p* = 0.63). On multivariate analysis, zinc deficiency (hazard ratio [HR] 1.83, *p* = 0.040) and prothrombin activity (HR 0.98, *p* = 0.042) were independent predictors of worse disease-free survival. Subgroup analysis revealed that Zn deficiency was significantly associated with elevated recurrence risk in patients with HCV-related liver disease (HR 3.67, *p* = 0.013). **Conclusions**: Preoperative Zn deficiency is an independent predictor of worse DFS after curative hepatectomy. Although further large-scale studies are required to confirm these findings, evaluating preoperative Zn status may help identify patients at high risk of HCC recurrence.

## 1. Introduction

Globally, hepatocellular carcinoma (HCC) remains a critical public health issue, ranking as the fifth most prevalent malignancy and the second primary cause of cancer-related mortality [1]. The worldwide incidence of this disease continues to climb, largely driven by underlying conditions including chronic viral hepatitis, non-alcoholic fatty liver disease, and alcohol-induced liver injury [2,3]. Despite ongoing advancements in diagnostic and therapeutic modalities, the management of HCC is heavily complicated by frequent post-resection recurrences [4]. Emerging research into HCC pathogenesis and novel treatment strategies continues to evolve, highlighting the persistent need for better predictive models [5,6]. Even following hepatectomy with curative intent, recurrence rates at five years remain substantial, often estimated between 50% and 80% [7,8,9]. Consequently, deploying effective strategies for early detection and timely intervention is essential to enhance the long-term prognosis of these patients [10,11]. Identifying reliable novel biomarkers capable of accurately stratifying recurrence risk and directing therapeutic choices is therefore a pressing clinical necessity [12].

Zinc (Zn) serves as an indispensable trace element, participating in more than 300 enzymatic processes that are fundamental to cellular operations, including DNA synthesis, immune system regulation, and the management of oxidative stress [13,14]. Zn deficiency has been linked to impaired immune function, chronic inflammation, and increased susceptibility to diseases such as diabetes, cardiovascular disorders, and certain cancers [15,16]. In oncology, abnormal serum Zn levels have been observed in various cancers, including breast, prostate, and gastric cancers [17,18]. Zn appears to inhibit carcinogenesis by stabilizing DNA, reducing oxidative damage, and promoting apoptosis, while Zn deficiency contributes to tumorigenesis through genomic instability and inflammatory pathways [19,20]. These multifaceted roles highlight Zn’s potential as both a biomarker and therapeutic target in disease management, including several cancers [21,22].

Previous studies have investigated the relationship between zinc status and HCC, with some suggesting a potential role for zinc in HCC development and progression [23,24]. These studies have explored the prognostic implications of Zn deficiency in patients with HCC, yielding mixed results [24]. Some studies have shown that low serum zinc levels are associated with worse prognosis in HCC patients [25,26,27]. On the other hand, another study found that serum Zn levels have no impact on the prognosis for patients with HCC [28]. However, these studies have limitations, including insufficient follow-up periods, a focus on viral hepatitis-induced HCC, and the use of strict criteria for Zn deficiency, which limit their generalizability. Furthermore, there is a paucity of data specifically examining the prognostic impact of zinc status in patients undergoing curative hepatectomy for HCC. Therefore, this study aimed to elucidate the prognostic significance of preoperative Zn deficiency in a cohort of patients with HCC undergoing initial curative hepatectomy, irrespective of the etiological factors contributing to HCC development. Specifically, we investigated the association between preoperative Zn deficiency and both disease-free survival (DFS) and overall survival (OS), utilizing the globally accepted cutoff value for Zn deficiency.

## 2. Materials and Methods

### 2.1. Study Design and Patient Selection

This study retrospectively analyzed patients who underwent first curative hepatectomy for hepatocellular carcinoma (HCC) between 1 January 2011 and 31 December 2018 (*n* = 150). Patients were excluded if they had a history of preoperative anticancer therapy or if comprehensive medical records necessary for accurate data analysis were absent (Supplementary Figure S1). The remaining patients (*n* = 105) were then categorized into two groups based on preoperative serum zinc levels: the Zinc Deficiency Group, defined as patients with preoperative Zn deficiency (<70 µg/dL) (*n* = 46), and the Normal Zinc Group, defined as patients without preoperative Zn deficiency (≥70 µg/dL) (*n* = 59). The present study design was approved by Asahikawa Medical University Research Ethics Committee (approval number: 23189).

### 2.2. Data Collection

Relevant clinicopathological data were systematically extracted from the electronic medical records of each patient. This included patient demographics such as age and gender. Laboratory findings included serum levels of Zn, alpha-fetoprotein (AFP), alanine aminotransferase, aspartate aminotransferase, total bilirubin, albumin, platelet count, prothrombin activity, white blood cell count, neutrophil count, lymphocyte count, and hemoglobin level. Serological markers of hepatitis B virus (HBV) infection (hepatitis B surface antigen [HBsAg]) and hepatitis C virus (HCV) infection (anti-HCV antibody [HCV-Ab]) were also collected. All laboratory parameters were analyzed at our institutional central laboratory. Specifically, serum zinc levels were measured using a colorimetric assay on an automated clinical chemistry analyzer. Routine biochemical tests were performed using established enzymatic or colorimetric methods in accordance with the Japan Society of Clinical Chemistry recommendations. Complete blood counts were analyzed using automated flow cytometry and electrical impedance methods, while prothrombin activity was evaluated via the light scattering method. Furthermore, tumor markers and viral serological markers were quantified using automated chemiluminescent immunoassay systems. Tumor characteristics included tumor size, tumor number (single vs. multiple), presence of vascular invasion, and histological differentiation (well-differentiated vs. others). Hepatic reserve function was assessed using the Child–Pugh classification [29]. Blood samples for laboratory tests, including serum zinc levels, were collected within one month prior to the scheduled hepatectomy.

### 2.3. Definitions

For the purposes of this analysis, Zn deficiency was established as a preoperative serum zinc level below 70 µg/dL, corresponding to widely recognized international criteria [30,31,32]. Recurrence of the tumor was identified either as the emergence of new HCC nodules in the residual liver or as distant metastases, verified via appropriate radiological modalities (such as computed tomography, magnetic resonance imaging, or ultrasound) or through the histopathological review of biopsied tissues. Disease-free survival (DFS) was measured from the day of the primary liver resection until the first documented evidence of tumor recurrence or patient death from any etiology. Overall survival (OS) represented the duration from the surgical intervention to mortality from any cause. Patients aged 65 years or older were defined as elderly. Considering its impact on prognosis, alpha-fetoprotein was categorized using a cutoff value of 100 ng/mL [33,34]. Etiology of HCC was determined based on serological markers of hepatitis B virus (HBV) infection (hepatitis B surface antigen) and hepatitis C virus (HCV) infection (anti-HCV antibody), as well as clinical history and imaging findings, to classify the underlying liver disease into the following categories: HBV, HCV, non-alcoholic steatohepatitis (NASH), alcoholic liver disease (ALD), and others (including coinfection with HBV and HCV and no underlying liver disease). Furthermore, for patients with HCV-related disease, sustained virological response (SVR) was defined as undetectable serum HCV RNA levels following the completion of antiviral therapy.

### 2.4. Surgery and Follow-Up

The surgical approach for each patient was individualized based on a comprehensive assessment of factors including tumor location, the extent of tumor invasion into surrounding tissues, the patient’s underlying liver function, and their overall clinical condition. This approach aligned with established guidelines and practices for HCC management [35,36]. Postoperative surveillance was conducted in accordance with our institutional protocol, which adhered to the recommendations outlined in the Clinical Practice Guidelines for Hepatocellular Carcinoma [35,36]. This protocol entailed regular clinical assessments, radiological imaging (including computed tomography, magnetic resonance imaging, or ultrasonography), and serum tumor marker measurements. These follow-up assessments were typically scheduled at 3- to 6-month intervals for the initial 2 years following surgery, and subsequently at 6- to 12-month intervals thereafter.

### 2.5. Statistical Analysis

Differences in continuous clinical data between the normal Zn and Zn-deficient cohorts were assessed via the Mann–Whitney U test, whereas categorical variables were evaluated utilizing Fisher’s exact test. To analyze survival outcomes (DFS and OS), Kaplan–Meier curves were constructed, with group comparisons executed using the log-rank test. Both univariate and multivariate Cox proportional hazards regression models were applied to determine independent prognostic indicators. The proportional hazards assumption was visually and statistically confirmed through the evaluation of Schoenfeld residuals, yielding no significant deviations. Additionally, Martingale residuals were utilized to confirm the linearity assumptions for continuous variables incorporated into the Cox models. Variables that yielded a *p*-value below 0.10 in the preliminary univariate evaluation were subsequently integrated into the multivariate models. Subgroup analyses were implemented to investigate outcome variations across different stratifications, including age (<65 vs. ≥65 years), sex, and background liver disease. There were no missing data for any of the variables in the final study cohort. All analytical procedures were carried out employing R software version 4.0.3 (R Foundation for Statistical Computing, Vienna, Austria), considering a two-tailed *p*-value of <0.05 as indicative of statistical significance.

## 3. Results

### 3.1. Baseline Characteristics and Comparison by Preoperative Zinc Status

The 105-patient cohort comprised 87 males (82.9%) and 18 females (17.1%), with a median age of 70 years (range, 44–84 years). The etiology of HCC varied, with 31 cases (29.5%) attributed to HBV infection, 30 cases (28.6%) to HCV infection, 17 cases (16.2%) to NASH, 14 cases (13.3%) to ALD, and 13 cases (12.4%) to other causes, including coinfection with HBV and HCV. Among the 30 patients with HCV-related disease, 11 (36.7%) had achieved SVR prior to surgery. Most patients (*n* = 102, 97.1%) were classified as Child–Pugh stage A, indicating well-preserved liver function. Regarding tumor characteristics, the median tumor size was 30 mm (range, 8–150 mm), and 96 patients (91.4%) presented with a solitary tumor. Vascular invasion was observed in 28 patients (26.7%), and 31 patients (29.5%) had well-differentiated HCC.

Patients were stratified into two groups based on their preoperative serum Zn levels: the Zn deficiency group (*n* = 46) with serum Zn levels below 70 µg/dL, and the normal Zn group (*n* = 59) with serum Zn levels of 70 µg/dL or higher. Regarding zinc supplementation, no patients received preoperative zinc therapy, and only 1 patient (2.2%) in the Zn deficiency group received oral zinc supplementation during the postoperative follow-up period. The detailed baseline characteristics of the overall cohort and the comparison between the two groups are summarized in Table 1. The Zn deficiency group exhibited significantly lower hemoglobin (13.4 vs. 14.1 g/dL, *p* = 0.001), lower prothrombin activity (82% vs. 91%, *p* = 0.015), lower serum albumin levels (4.0 vs. 4.4 g/dL, *p* < 0.001), and smaller tumor size (26.5 vs. 35.0 mm, *p* = 0.008), compared with the normal Zn group. Notably, no significant differences were observed between the two groups in terms of age, sex, body mass index, etiology of HCC, or the presence of vascular invasion.

Continuous variables were analyzed using the Mann–Whitney U test, and categorical variables were analyzed using Fisher’s exact test.

### 3.2. Survival Analysis

Kaplan–Meier survival analysis was performed to evaluate the impact of preoperative Zn deficiency on postoperative outcomes. The median follow-up duration was 62.7 months (range, 1–140.5 months). Complete follow-up data were available for all 105 included patients, and no patients were lost to follow-up during the observation period. Among the 54 patients who experienced tumor recurrence, 51 (94.4%) subsequently underwent active salvage therapies, including repeat hepatectomy, transarterial chemoembolization, or systemic therapies. During the observation period, 33 patients died (15 in the Zn deficiency group and 18 in the normal Zn group). Regarding the causes of mortality, non-HCC-related deaths (such as liver failure, pneumonia, or other comorbidities) accounted for 50.0% (9 out of 18) of the deaths in the normal Zn group, compared to only 20.0% (3 out of 15) in the Zn deficiency group. The Zn deficiency group showed a significantly worse DFS compared to the normal Zn group (log-rank *p* = 0.015) (Figure 1a). The 1-, 3-, and 5-year DFS rates were 78.3%, 50.9%, and 33.7%, respectively in the Zn deficiency group, while those in the normal Zn group were 90.3%, 74.1%, and 56.5%, respectively. However, there was no statistically significant difference in OS between the two groups (log-rank *p* = 0.63) (Figure 1b).

### 3.3. Univariate and Multivariate Analyses

To identify independent predictors of DFS and OS, univariate and multivariate Cox proportional hazards regression analyses were conducted. Covariates with a *p*-value < 0.10 in the univariate analysis were included in the multivariate analysis. Regarding DFS, the multivariate analysis (Table 2) identified preoperative Zn deficiency (HR 1.78, 95% CI: 1.03–3.08, *p* = 0.039) and low prothrombin activity (HR 0.98, 95% CI: 0.96–1.00, *p* = 0.042) as independent unfavorable prognostic factors. Conversely, regarding OS, the multivariate analysis (Table 3) demonstrated that a well-differentiated tumor was the only independent favorable prognostic factor for overall survival (HR 0.22, 95% CI: 0.05–0.96, *p* = 0.043). Further supplementary analysis within this HCV subgroup revealed no statistically significant differences between patients with and without SVR regarding DFS (45.5% [5/11] vs. 68.4% [13/19], *p* = 0.266) and the prevalence of preoperative zinc deficiency (18.2% [2/11] vs. 47.4% [9/19], *p* = 0.140).

### 3.4. Subgroup Analysis

To further explore the impact of Zn deficiency on DFS in different patient subgroups, we performed subgroup analyses based on age (<65 vs. ≥65 years), gender (male vs. female), and etiology of liver disease (HBV, HCV, NASH, ALD, and other). The results of the subgroup analyses are presented in Figure 2. Zn deficiency was significantly associated with worse DFS in patients aged 65 years or older (HR 2.08, 95% CI: 1.10–3.94, *p* = 0.024) and male patients (HR 2.07, 95% CI: 1.13–3.79, *p* = 0.018). Regarding etiology, a significant association between Zn deficiency and DFS was also observed in patients with HCV-related liver disease (HR 3.67, 95% CI: 1.32–10.17, *p* = 0.013).

## 4. Discussion

This study investigated the prognostic impact of preoperative Zn deficiency in patients with HCC undergoing curative hepatectomy. Our findings demonstrate that preoperative Zn deficiency is significantly associated with worse DFS, particularly in patients with HCV-induced HCC. This result suggests that preoperative Zn status might serve as a novel prognostic marker to identify patients with HCC at high risk of recurrence after hepatectomy.

We found that hemoglobin, platelet, and prothrombin activity were lower in the Zn deficiency group, aligning with previous reports [25,26,37,38]. Meanwhile, tumor-related factors did not differ significantly between the two groups, and tumor size was rather smaller in the Zn deficiency group. These results suggested that the higher recurrence rate in Zn-deficient patients is not attributed to a more malignant tumor trait leading to metastatic recurrence of the resected HCC, but rather points to the likelihood of metachronous multicentric occurrences, potentially driven by a pro-oncogenic cirrhotic microenvironment exacerbated by zinc deficiency [39]. Furthermore, serum Zn has been shown to correlate with various markers of liver function and fibrosis, serving as a useful indicator of hepatic damage and the degree of fibrosis [25,40,41]. Some evidence indicated that Zn was more sensitive than conventional liver function parameters—including the Child–Pugh classification—in detecting subclinical hepatic dysfunction [26,42,43]. Early recognition of Zn deficiency might therefore help identify patients at higher risk of postoperative HCC recurrence and allow for timely interventions aimed at improving long-term outcomes.

Our finding that preoperative Zn deficiency is associated with worse postoperative DFS in HCC is partially consistent with previous studies in terms of prognosis, but there are some differences. Imai et al. [25] and Harimoto et al. [27] showed that Zn deficiency was an independent predictor of worse OS in patients with HCC after liver resection, but not in DFS. In contrast, in our study, Zn deficiency was an independent prognostic factor for worse DFS, although there was no significant difference in OS between Zn deficiency and normal Zn groups. These discrepancies can be explained by differences in study eras, threshold definitions, patient backgrounds (particularly the prevalence of HCV infection), and post-recurrence clinical courses. Imai et al. analyzed an older cohort (2004–2012), reflecting an era before modern, highly active salvage therapies were widely available, making OS more directly dependent on initial recurrence. Conversely, while Harimoto et al. analyzed a recent cohort, their use of a stringent cut-off (<60 µg/dL) likely selected patients with profound liver dysfunction. In such cases, the observed OS difference might have been driven primarily by direct liver-related mortality rather than HCC recurrence. Furthermore, the proportion of HCV-related HCC differed substantially across the studies (48.4% in Imai et al., 39.7% in Harimoto et al., and 28.6% in our cohort). Given that our subgroup analysis demonstrated a significant prognostic impact of Zn deficiency on DFS within the HCV cohort, these etiological differences likely contributed to the divergent survival outcomes observed. Our cohort, characterized by well-preserved liver function (97.1% Child–Pugh A) and a globally accepted threshold (<70 µg/dL), specifically captured the subclinical deficiency that promotes oncogenesis (DFS impact) prior to overt liver failure. In our study, the overwhelming majority of patients who experienced recurrence (94.4%) underwent intensive, multimodal salvage therapies, effectively prolonging their OS. These aggressive post-recurrence interventions, coupled with higher competing mortality in the normal Zn group, likely obscured the impact of preoperative Zn status on OS. Studies in a broader population of patients with HCC, including those treated with non-surgical therapies, have reported conflicting results regarding whether zinc concentrations are associated with prognosis in HCC [26,28]. These discrepancies underscore the need for further comprehensive, large-scale studies to clarify the complex relationship between zinc and postoperative HCC prognosis.

The mechanisms underlying the association between Zn deficiency and worse DFS in HCC patients remain unclear. However, several potential explanations can be proposed. Zn plays a critical role in various cellular processes, including DNA synthesis, repair, and cell cycle regulation [44,45]. Zn deficiency impairs these processes, leading to genomic instability and increased susceptibility to carcinogenesis [46,47,48]. Additionally, Zn has been shown to modulate immune function and inhibit angiogenesis, both of which are critical in HCC development and progression [49,50]. Zn deficiency compromises immune surveillance and promotes tumor angiogenesis, thereby facilitating tumor growth and metastasis [20,24,51].

Our subgroup analysis revealed that a significant association between Zn deficiency and worse DFS was also observed in patients with HCV-induced HCC, consistent with previous reports [25]. Zn has multifaceted effects on viral replication, oxidative stress, and immune response in patients with HCV infection, potentially explaining the especially detrimental impact of zinc deficiency in this subgroup. Several studies have suggested that Zn and Zn derivatives inhibited HCV replication [52,53,54]. Furthermore, the NS3 protein domain of HCV is a Zn-containing enzyme [55], and the NS5A protein is a Zn metalloprotein [56]. Both proteins play critical roles in HCV replication, and Zn is essential for their structural stability and function [57,58]. Introducing mutations into their zinc-binding sites impairs HCV replication [56,59], indicating that Zn is an indispensable factor utilized in HCV replication and influence the homeostasis of serum Zn. Furthermore, HCV infection triggers oxidative stress in hepatocytes—potentially causing mitochondrial damage—and Zn plays a crucial role in mitigating this process by activating antioxidative enzymes (e.g., superoxide dismutase) and inhibiting lipid peroxidation [60,61]. Consequently, Zn deficiency can amplify oxidative damage and accelerate the progression of liver fibrosis and HCC [39]. Additionally, Zn is pivotal for maintaining normal immune function, influencing T-cell activation, cytokine production, and broader immune defenses [62]. Thus, inadequate Zn status might weaken antiviral immunity, foster chronic HCV infection and exacerbate liver disease progression [63]. In our cohort, 11 of the 30 HCV patients (36.7%) had achieved SVR prior to surgery, and postoperative Zn supplementation was rarely administered. Given the limited sample size, the precise prognostic impact of uncorrected Zn deficiency following SVR, as well as the potential efficacy of postoperative Zn supplementation [61,62], remain unclear and warrant further investigation.

This study has several limitations. Its retrospective design introduces the potential for selection bias and unmeasured confounding factors. The relatively small sample size and single-center nature of the study might limit the generalizability of our findings to other populations. Furthermore, the limited number of events within specific patient subgroups precluded the execution of formal statistical interaction tests. Additionally, we did not assess dietary zinc intake, which could provide a more complete picture of each patient’s zinc status. To address these limitations and further clarify the role of zinc in the prognosis of HCC, further prospective studies with larger sample sizes and detailed assessment of zinc intake at multiple centers are needed.

## 5. Conclusions

In conclusion, our study demonstrated that preoperative Zn deficiency is an independent prognostic factor for worse DFS in patients with HCC undergoing curative hepatectomy. This finding suggests that preoperative zinc status may serve as a potential adjunctive marker to identify patients at high risk of recurrence, allowing for more tailored postoperative surveillance strategies. However, given the lack of an overall survival benefit in this cohort, prospective multi-center studies are required to fully establish its clinical utility in therapeutic decision-making.

## Figures and Tables

**Figure 1:**
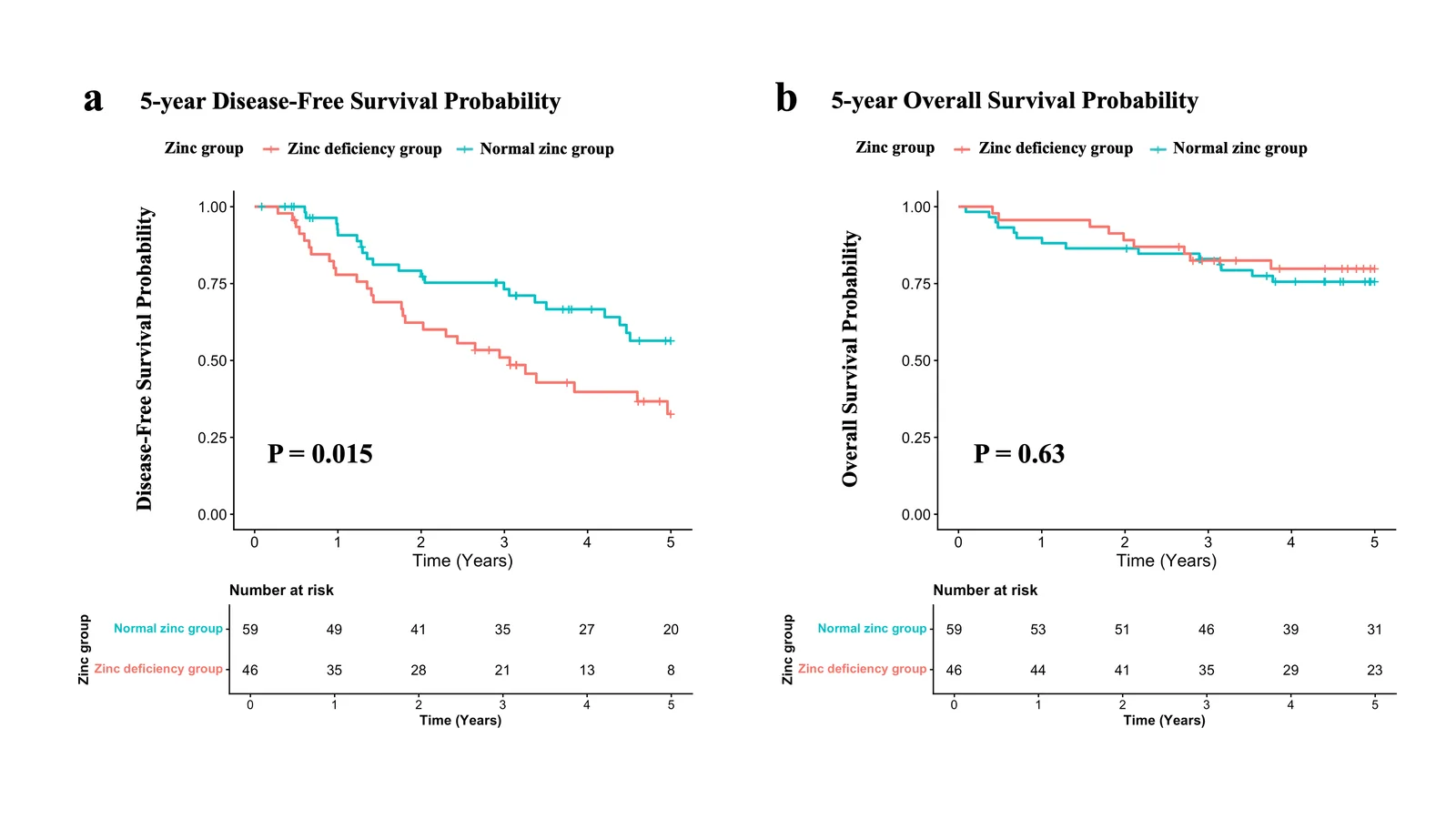
Comparison of Kaplan–Meier curves for disease-free survival and overall survival by preoperative serum zinc status in patients who underwent curative hepatectomy for hepatocellular carcinoma. (**a**) Disease-free survival and (**b**) overall survival in Zn deficiency (red line) and normal Zn groups (blue line) are shown.

**Figure 2:**
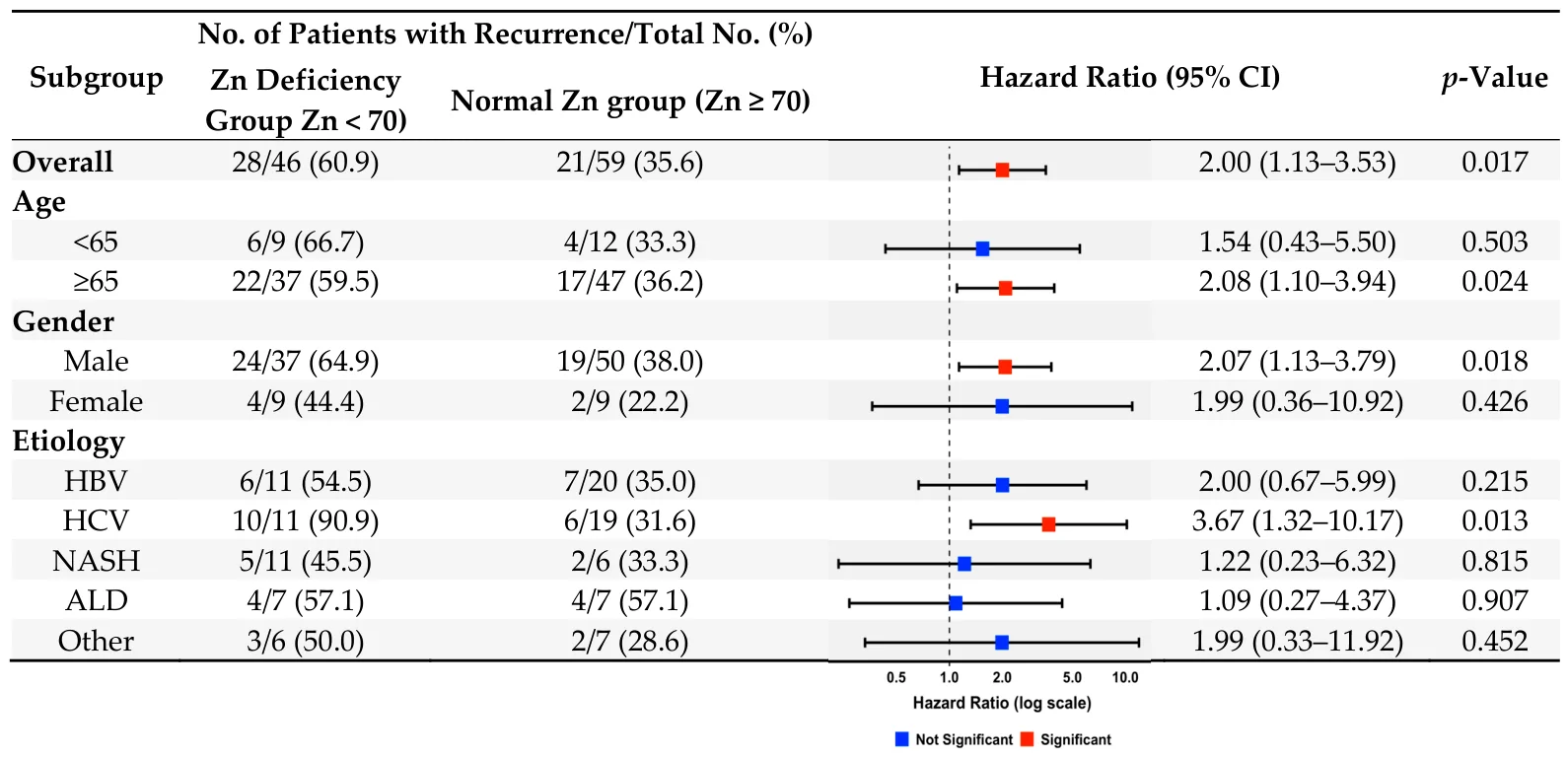
Subgroup analysis for postoperative disease-free survival in patients who underwent curative hepatectomy for hepatocellular carcinoma.

**Table 1:** Baseline patient characteristics and comparison between the Zn deficiency and normal Zn groups.

Variable	Median (Min–Max)/Count			*p*-Value
	Overall (*n* = 105)	Zn Deficiency Group <70 (*n* = 46)	Normal Zn Group ≥70 (*n* = 59)	
Age (years)	70 (44–84)	70 (46–84)	71 (44–84)	0.905
Gender (Male/Female)	87/18	37/9	50/9	0.608
BMI (kg/m^2^)	23.6 (14.1–32.2)	23.6 (14.1–31.14)	23.6 (16.6–32.2)	0.851
Etiology (HBV/HCV/NASH/ALD/Other)	31/30/17/14/13	11/11/11/7/6	20/19/6/7/7	0.316
HBs-Ag (Positive/Negative)	32/73	11/35	21/38	0.209
HCV-Ab (Positive/Negative)	31/74	11/35	20/39	0.289
White blood cell count (10^3^/μL)	5.14 (2.57–12.61)	4.92 (2.92–12.61)	5.23 (2.57–9.23)	0.905
Neutrophil count (10^3^/μL)	3.17 (1.45–10.95)	3.09 (1.54–10.95)	3.34 (1.45–6.36)	0.854
Lymphocyte count (10^3^/μL)	1.28 (0.45–3.47)	1.26 (0.47–3.32)	1.34 (0.64–3.47)	0.118
Hemoglobin (g/dL)	13.7 (8.6–20.7)	13.4 (9–17)	14.1 (8.6–20.7)	0.001
Platelet (10^3^/μL)	156 (49–453)	138.5 (49–372)	160 (73–453)	0.080
Prothrombin activity (%)	89 (28–127)	82 (32.3–122)	91 (28–127)	0.015
AST (IU/L)	33.0 (14–121)	31 (18–111)	33 (14–121)	0.774
ALT (IU/L)	28.0 (8–132)	28 (12–132)	28 (8–122)	0.846
Total bilirubin (mg/dL)	0.8 (0.1–2.1)	0.8 (0.3–2.1)	0.8 (0.1–1.6)	0.772
Zn (µg/dL)	71 (44–99)	63.5 (44–69)	78 (70–99)	<0.001
Albumin (g/dL)	4.2 (2.8–5.1)	4.0 (2.8–4.8)	4.4 (3.4–5.1)	<0.001
Child-Pugh (A/B/C)	102/3/0	43/3	59/0	0.081
AFP (<100/≥100 ng/mL)	87/18	39/7	48/11	0.795
Tumor number (Single/Multiple)	96/9	40/6	56/3	0.175
Tumor size (mm)	30 (8–150)	26.5 (10–120)	35 (8–150)	0.008
Vascular invasion (Negative/Positive)	28/77	36/10	41/18	0.377
Differentiation (well/other)	31/74	15/31	16/43	0.667
pStage (1/2/3/4)	55/24/19/7	25/9/9/3	30/15/10/4	0.936

HBV: hepatitis B virus; HCV: hepatitis C virus; NASH: non-alcoholic steatohepatitis; ALD: alcoholic liver disease; AFP: alpha-fetoprotein; AST: aspartate transaminase; ALT: alanine aminotransferase; BMI: body mass index; HBs-Ag: hepatitis B surface antigen; HCV-Ab: anti-hepatitis C antibody; Zn: zinc.

**Table 2:** Cox regression analysis prognostic factors for postoperative disease-free survival in patients who underwent curative hepatectomy for hepatocellular carcinoma.

Variables	Univariate Analysis		Multivariate Analysis	
	Hazard Ratio (95% CI)	*p*-Value	Hazard Ratio (95% CI)	*p*-Value
Age (years)	1.01 (0.98–1.04)	0.631		
Gender (Male)	1.79 (0.76–4.21)	0.182		
HBs-Ag (Positive)	0.77 (0.41–1.42)	0.399		
HCV-Ab (Positive)	1.36 (0.75–2.47)	0.314		
Platelet count (10^3^/μL)	1.00 (0.99–1.00)	0.195		
Prothrombin activity (%)	0.98 (0.96–1.00)	0.016	0.98 (0.96–1.00)	0.042
AST (IU/L)	1.01 (0.99–1.02)	0.344		
ALT (IU/L)	1.01 (1.00–1.02)	0.057	1.01 (0.99–1.02)	0.125
Albumin (g/dL)	0.86 (0.46–1.60)	0.631		
Zn (<70 µg/dL)	2.00 (1.13–3.53)	0.017	1.83 (1.03–3.25)	0.040
Child-Pugh (B)	2.05 (0.49–8.52)	0.324		
AFP (≥100 ng/mL)	0.80 (0.36–1.78)	0.581		
Tumor number (Multiple)	1.41 (0.56–3.56)	0.468		
Tumor size (mm)	0.63 (0.28–1.40)	0.258		
Vascular invasion (Positive)	0.55 (0.26–1.16)	0.116		
Differentiation (Well)	0.71 (0.38–1.34)	0.291		

AFP: alpha-fetoprotein; AST: aspartate transaminase; ALT: alanine aminotransferase; HBs-Ag: hepatitis B surface antigen; HCV-Ab: anti-hepatitis C antibody; Zn: zinc.

**Table 3:** Cox regression analysis prognostic factors for postoperative overall survival in patients who underwent curative hepatectomy for hepatocellular carcinoma.

Variables	Univariate Analysis		Multivariate Analysis	
	Hazard Ratio (95% CI)	*p*-Value	Hazard Ratio (95% CI)	*p*-Value
Age (years)	1.02 (0.97–1.07)	0.524		
Gender (Male)	4.83 (0.65–35.83)	0.124		
HBs-Ag (Positive)	0.58 (0.21–1.55)	0.275		
HCV-Ab (Positive)	1.03 (0.42–2.51)	0.945		
Platelet count (10^3^/μL)	1.01 (1.00–1.01)	0.041	1.00 (0.99–1.01)	0.136
Prothrombin activity (%)	1.00 (0.97–1.03)	0.917		
AST (IU/L)	1.00 (0.99–1.02)	0.569		
ALT (IU/L)	1.00 (0.99–1.02)	0.847		
Albumin (g/dL)	0.84 (0.34–2.04)	0.699		
Zn (<70 µg/dL)	0.81 (0.35–1.88)	0.627		
Child-Pugh (B)	1.89 (0.25–14.06)	0.535		
AFP (≥100 ng/mL)	1.82 (0.72–4.63)	0.205		
Tumor number (Multiple)	1.58 (0.47–5.31)	0.461		
Tumor size (mm)	2.18 (0.92–5.14)	0.075	1.27 (0.48–3.30)	0.627
Vascular invasion (Positive)	2.03 (0.88–4.70)	0.097	1.49 (0.59–3.74)	0.398
Differentiation (Well)	0.21 (0.05–0.90)	0.036	0.22 (0.05–0.96)	0.043

AFP: alpha-fetoprotein; AST: aspartate transaminase; ALT: alanine aminotransferase; HBs-Ag: hepatitis B surface antigen; HCV-Ab: anti-hepatitis C antibody; Zn: zinc.

## Data Availability

The data presented in this study are available on request from the corresponding author. The data are not publicly available due to strict privacy and ethical restrictions regarding patient confidentiality.

## Supplementary Materials

The following supporting information can be downloaded at https://www.mdpi.com/article/10.3390/nu18193147/s1, Supplementary Figure S1: Study Design and Patient Selection.

## Abbreviations

The following abbreviations are used in this manuscript:

ALD	Alcoholic liver disease
DFS	Disease-free survival
HBsAg	Hepatitis B surface antigen
HBV	Hepatitis B virus
HCC	Hepatocellular carcinoma
HCV	Hepatitis C virus
HCV-Ab	Anti-hepatitis C virus antibody
HR	Hazard ratio
NASH	Non-alcoholic steatohepatitis
OS	Overall survival
SVR	Sustained virological response
Zn	Zinc
