# Supplementary material for: Preoperative Zinc Deficiency as a Novel Prognostic Predictor for Postoperative Recurrence in Hepatocellular Carcinoma
Source: Nutrients. 2026 Sep 24;18(19):3147. doi: 10.3390/nu18193147 (PMC13666986; doi:10.3390/nu18193147)

# Supplementary Figure S1

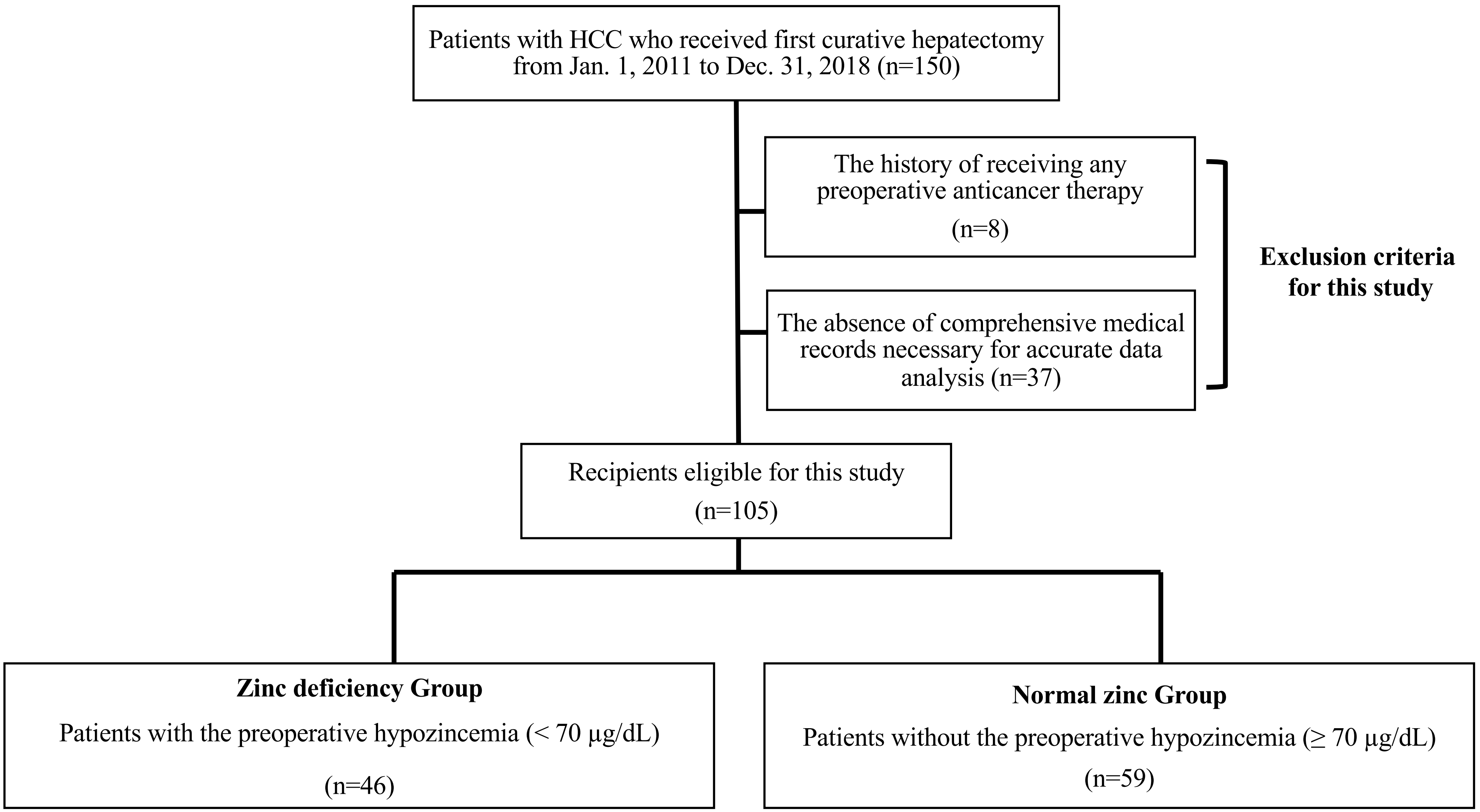

Supplement: Supplementary file 1 [file nutrients-18-03147-s001.zip › nutrients-4491094-supplementary.pdf]
